# The relationship between ethnic composition of the residential environment and self-reported health among Turks and Moroccans in Amsterdam

**DOI:** 10.1186/s12942-017-0084-x

**Published:** 2017-04-12

**Authors:** Eleonore M. Veldhuizen, Umar Z. Ikram, Sjoerd de Vos, Anton E. Kunst

**Affiliations:** 1 Department of Human Geography, Planning and International Development Studies, Faculty of Social and Behavioural Sciences, University of Amsterdam, Nieuwe Achtergracht 166, 1018 WV Amsterdam, The Netherlands; 2 Department of Public Health, Academic Medical Centre, University of Amsterdam, Meibergdreef 9, 1105 AZ Amsterdam, The Netherlands

**Keywords:** Neighbourhood ethnic composition, Ethnic density, Ethnic heterogeneity, Self-reported health, Spatial scale, Geographically weighted regression

## Abstract

**Background:**

Previous studies from the US and UK suggest that neighbourhood ethnic composition is associated with health, positive or negative, depending on the health outcome and ethnic group. We examined the association between neighbourhood ethnic composition and self-reported health in these groups in Amsterdam, and we aimed to explore whether there is spatial variation in this association.

**Methods:**

We used micro-scale data to describe the ethnic composition in buffers around the home location of 2701 Turks and 2661 Moroccans. Multilevel regression analysis was used to assess the association between three measures of ethnic composition (% co-ethnics, % other ethnic group, Herfindahl index) and three measures of self-reported health: self-rated health, Physical and Mental Component Score (PCS, MCS). We adjusted for socioeconomic position at individual and area level. We used geographically weighted regression and spatially stratified regression analyses to explore whether associations differed within Amsterdam.

**Results:**

Ethnic heterogeneity and own ethnic density were not related to self-rated health for both ethnic groups. Higher density of Turks was associated with better self-rated health among Moroccans at all buffer sizes, with the most significant relations for small buffers. Higher heterogeneity was associated with lower scores on PCS and MCS among Turks (suggesting worse health). We found spatial variation in the association of the density of the other ethnic group with self-rated health of Moroccans and Turks. We found a positive association for both groups, spatially concentrated in the sub-district Geuzenveld.

**Conclusions:**

Our study showed that the association of ethnic composition with self-reported health among Turks and Moroccans in Amsterdam differed between the groups and reveals mainly at small spatial scales. Among both groups, an association of higher density of the other group with better self-rated health was found in a particular part of Amsterdam, which might be explained by the presence of a relatively strong sense of community between the two groups in that area. The study suggests that it is important to pay attention to other-group density, to use area measurements at small spatial scales and to examine the spatial variation in these associations. This may help to identify neighbourhood characteristics contributing to these type of area effects on urban minority health.

## Background

European societies have become increasingly ethnically diverse over the last decades, and this demographic shift is likely to continue given the relatively high influx of immigrants [1, 2]. Evidence indicates that ethnic minority groups overall tend to have worse self-rated health than the ethnic majority group in European countries [3]. This has been attributed to low individual socioeconomic status (SES) and psychosocial factors (e.g., discrimination, acculturation, social network) [4–7], amongst other factors.

Contextual factors such as characteristics of the residential environment may also shape the health of ethnic minority groups. One such characteristic is ethnic composition, which is conceptualized as ethnic diversity or as own-group density (i.e., the presence of the same-ethnic group in the residential environment) [8]. The association between ethnic composition of the residential environment and health presumably operates through social capital and exposure to discrimination [9]. However, evidence from the United States (US) and Europe is equivocal, in that the strength and direction (both negative and positive) of the association vary by ethnic minority group, spatial scale, and outcome measure [8–12].

Furthermore, the existing literature on this topic has three potential limitations. First, most epidemiological studies have focused on own-group density or ethnic diversity, while relatively few studies have assessed other-group density (i.e., co-presence of a specific other ethnic group). This might be particularly relevant for some cities in which two or more (large-sized) ethnic minority groups reside. Other-group density might affect health through material and psychosocial processes. The association could be either positive or negative, largely depending on the inter-relationship the groups have (e.g. mutual trust, discrimination, sharing job information) [13–15].

Second, previous studies have used large spatial scales (e.g., census tracts, electoral wards), making it potentially difficult to assess the associations with health outcomes accurately [16]. Most inter-ethnic interaction and the underlying material and psychosocial processes are likely to occur at smaller spatial scales in the direct environment. Hence using smaller spatial scales could possibly better capture the associations between ethnic composition of residential environment and health [16].

Third, most studies have presented the aggregated effects of ethnic composition on health at city-level [8–11]. This may possibly obscure the spatial variation within a city. Different parts of a city may differ in the opportunities they provide for social interaction between groups. These opportunities might be different due to differences in physical environments (e.g., built environment) and social environments (e.g., social cohesion, local institutions) [16]. So far, it is unknown whether the association between ethnic composition of residential environment and health differs within a city.

In the present study, we aimed to fill these gaps in the literature. First, we aimed to investigate the association between other-group density of the residential environment and self-reported health outcomes in two ethnic minority groups. We further considered other measures of ethnic composition of residential environment: ethnic heterogeneity and own-group density. Second, we assessed the associations at different spatial scales (both small and large). Third, we explored spatial variation, by assessing whether the associations differed within the city.

We focused on Turkish and Moroccan adults residing in Amsterdam, the Netherlands. These two groups are considered the largest ethnic minority groups in Europe, and tend to co-exist in many different cities (e.g. Paris, Berlin). Our study extended previous studies on this topic conducted in Amsterdam. A 2014 study found own-group density was not associated with psychological distress in Turkish and Moroccan adults living in the four largest Dutch cities (including Amsterdam) [12]. A more recent study from Amsterdam suggested that a high density of Moroccan residents was associated with poor self-rated health among Turkish residents, but not vice versa [13]. In the present study, we delve into these findings by using a much larger dataset, more health outcomes and different spatial scales, as well as by assessing variation within the city.

## Study population and methods

### Study population

The data were obtained from the HELIUS (Healthy Life in an Urban Setting) study. The aims and design of the HELIUS study have been described elsewhere [17]. Briefly, HELIUS is a large-scale cohort study on health and healthcare among different ethnic groups living in Amsterdam. It included individuals aged 18–70 years from the six largest ethnic groups living in Amsterdam, i.e. those of Dutch, South-Asian Surinamese, African Surinamese, Ghanaian, Moroccan and Turkish origin. Participants were randomly sampled from the municipal registers, stratified by ethnicity. Data were collected by questionnaire and a physical examination. At the end of 2014, response rates were estimated between 20 and 40% with some variations across ethnic groups.

For the current study, baseline data collected from January 2011 until December 2014 were used, including 2962 Turkish and 3000 Moroccan participants. Individuals with missing data on self-reported health, individual characteristics, area ethnic composition or area socioeconomic position, and individuals living at locations with <25 inhabitants within a buffer of 50 m were excluded from the analysis (n = 600). Our final sample comprised 5362 participants: 2701 Turks and 2661 Moroccans.

### Individual level measurements

Participant’s ethnicity was defined according to the country of birth of the participant as well as that of his/her parents. Specifically, a participant is considered of Turkish/Moroccan origin if: (1) he or she was born in Turkey/Morocco and has at least one parent born in Turkey/Morocco; or (2) he or she was born in the Netherlands but both his/her parents born in Turkey/Morocco [18].

Three measures of self-reported health are used: self-rated health and generic physical and mental health (PCS and MCS). Self-rated health was measured by the response to the question, ‘In general, would you say your health is excellent, very good, good, fair or poor?’ The answers were classified into two categories: excellent/very good/good and fair/poor. In the remainder of the paper we refer to the first category as better self-rated health. Generic mental and physical health were assessed using the component summary measures of physical (PCS) and mental health (MCS) from the Medical Outcomes Study Short Form 12 (SF-12) [19]. Scores range from 0 to 100 with higher scores reflecting better health.

From the same survey, we obtained data on characteristics of the participants that were used as control variables at the individual level. These include age, sex, marital status, household composition, educational level, length of residence in the country and a measure of general wealth (whether the participant experienced difficulties living on his or her current household income). See Table 1 for a description of these variables.

**Table 1:** Characteristics of participants and their socio-economic environment, per ethnic group

Ethnic group	Moroccan	Turkish
*N*	2661	2701
Self-rated health (%)		
Excellent	4.7	4.0
Very good	9.7	10.8
Good	48.1	49.6
Fair	30.5	26.4
Poor	7.1	9.3
Physical Component Score		
Mean	46.0	45.3
Standard dev	10.2	10.7
Mental Component Score		
Mean	46.1	44.8
Standard dev	10.9	11.3
Length of residence in the country (years)		
Mean	28.6	28.2
Standard dev	8.7	8.2
Age (%)		
18–29	28.3	25.0
30–39	22.3	21.1
40–49	22.4	29.5
50–64	24.4	22.7
≥65	2.6	1.7
Sex (%)		
Male	36.6	46.3
Marital status (%)		
Married couple	57.6	62.6
Unmarried couple	2.3	3.4
Never been married	28.4	21.5
Divorced	10.1	10.2
Widow/widower	1.7	2.3
Household composition (%)		
Single	7.2	9.2
Couple without children	7.3	10.3
Family	49.2	52.1
Other (living with parents, parents in law, institution)	36.3	28.4
Education (%)		
No/elementary	33.1	33.3
Lower secondary	18.0	25.5
Intermediate/higher secondary	33.1	27.5
Higher	15.9	13.6
Living on household income (%)		
No problems at all	22.3	16.8
No problems, but I have to watch what I spend	35.6	25.4
Some problems	26.3	31.3
Lots of problems	15.7	26.5
Property value of houses at postcode of residence (€)		
Mean	198,216	193,880
Standard dev	55,915	53,692
% Households living on a minimum income at postcode of residence		
Mean	28.4	25.9
Standard dev	14.4	15.5

### Area-level measurements

For area-level measurements we used integral demographic and socio-economic registries at the level of full 6-digit postcodes maintained by the Department of Research and Statistics of the Municipality of Amsterdam. Data on the spatial level of 6-digit postcode area is the most detailed data available. On average, these units are sized 50 × 50 m and include 10–20 households.

To describe the ethnic composition for each participant, we constructed three variables: *own*-*group density* (i.e., percentage of co-ethnics), *other*-*group density* (i.e., percentage of the other ethnic group—Turks or Moroccans) and *ethnic heterogeneity* described by the Herfindahl-index. This index yields the probability of two randomly selected individuals from the same neighbourhood being of different ethnic origin. The theoretical range of the index runs from 0 to 1, with 0 representing an area in which every individual is from the same ethnic group and 1 representing an area in which every individual is from a different ethnic group. To calculate this index, we sum the squared proportion of each ethnic group (Surinamese, Antilleans, Ghanaians, Turks, Moroccan, other non-western migrants, other western migrants and Dutch) and subtract this total from one.

When studying the association between ethnic composition and health, it is not enough to control for individual characteristics only. Veldhuizen et al. [13] showed that it is necessary to control for the socio-economic environment as well, because this variable can act as a confounder. To describe the socio-economic environment we constructed two socio-economic variables: the percentage of residents living on a minimum income and the average property value of houses.

In general, the multicollinearity between the independent variables is not very high. Most correlations are 0.5 at most. Only the correlations between percentage Turks/percentage Moroccans/heterogeneity (Herfindahl index) and percentage of minimum income households are high for the larger buffers (0.7). However, because socioeconomic environment is an important determinant of self-rated health we cannot remove the variable from our model.

Within a Geographical Information System (ArcGIS) we created buffers of varying sizes, with radiuses ranging from 50 to 1000 m, around the central point location of each participant’s 6-digit postcode area. The ethnic composition and socioeconomic characteristics of each of these buffers were estimated by aggregating the postcode data to the buffers. For a more detailed description of the procedure see Veldhuizen et al. [20].

### Statistical analysis

The associations between ethnic composition of the residential environment and self-rated health were assessed using multilevel logistic regression analysis, with better self-rated health as the dependent variable and 6-digit postcode as the variable indicating the higher level (participants living in the same postcode area have identical buffers). We adjusted for the individual characteristics age, sex, marital status, household composition, education, length of residence in the country and wealth and for socio-economic environment measured by the percentage of households living on minimum income and average property value.

To enable comparison of the results of these analyses between different predictors and the different buffer sizes, we present standardised odds ratios of the three measures of ethnic composition. These odds ratios can be interpreted as the change in the odds of better self-rated health if a predictor variable increases with one standard deviation. The odds ratios take into account the differences in standard deviation according to predictor and buffer size (Table 2).

**Table 2:** Characteristics of the participant’s neighbourhood ethnic composition per ethnic group and spatial scale

Ethnic group	Moroccan		Turkish	
	Mean	SD	Mean	SD
Own ethnic density (%)				
Buffer50	26.6	16.5	17.2	10.5
100	23.5	14.9	15.0	8.6
150	22.2	14.1	14.3	7.9
300	19.7	11.7	12.9	6.8
500	18.3	10.3	12.2	6.3
750	17.2	9.0	11.7	6.0
1000	16.3	8.3	11.3	5.9
Other ethnic density (%) (Turks resp. Moroccans)				
50	12.6	9.9	22.9	15.3
100	12.3	8.6	22.6	13.4
150	12.0	8.1	22.2	12.6
300	11.1	7.1	20.7	10.5
500	10.5	6.5	19.7	9.0
750	10.0	6.1	18.7	8.0
1000	9.6	5.9	18.0	7.5
Ethnic heterogeneity (range 0–1)				
50	0.711	0.085	0.722	0.083
100	0.721	0.079	0.735	0.072
150	0.723	0.079	0.738	0.070
300	0.728	0.078	0.744	0.066
500	0.729	0.077	0.747	0.064
750	0.728	0.075	0.745	0.063
1000	0.725	0.073	0.742	0.062

The associations between neighbourhood ethnic composition and PCS and MCS were assessed using multilevel linear regression analysis, adjusting for the same individual and environmental variables as mentioned above. We present standardised regression coefficients. These coefficients can be interpreted as the change in the standardised dependent variable in case the predictor variable increases with one standard deviation.

In total, 2251 postcode areas were included in the analysis; 1507 for the Turks and 1572 for the Moroccans. We applied random effects (intercept) estimators using STATA’s melogit and mixed commands. Random effects appeared to be significant in all empty models and in approximately half of the models with variables. Because a significant number of postcode areas include only one or a limited number of participants, it was not possible to accurately measure both variations between and within the areas. As a result, likelihood ratio tests indicated that our random intercept models were not statistically significant in several models, implying limited meaning of random effects models compared to models without random effects. We present the parameters of the multilevel models because these models generated greater standard errors for our variables of interest than models without random effects.

The dependent variables show substantial variation over Amsterdam. For instance, across 22 administratively defined areas, for Turks the percentage of participants with good self-rated health varies between 44 and 77, for Moroccans between 50 and 73.

### Geographical analysis

Additionally, we used logistic geographically weighted regression (GWR) within the software GWR4 to explore whether the most important association we found from the multilevel regression analyses spatially differed within Amsterdam. GWR enables us to explore if the association varies within the city, without a priori assumptions with respect to the geographic scale at which these variations would occur. GWR is a local form of (in this case logistic) regression to model spatially varying relationships. It constructs a separate equation for every participant incorporating the dependent and explanatory variables of all participants living within a specific distance around the target participant. We used a bandwidth (Gaussian Kernel) of a fixed distance of 500 m which means that a 500 m kernel is used over the whole study area. The alternative for a fixed spatial kernel, an adaptive kernel, varies the size of kernel according to the spatial distribution of observation. This would mean that in areas with relatively few participants the kernel would become large which would obscure local relationships. We considered 500 m as a reasonable compromise between two conflicting demands: (1) to include a reasonable number of participants in the analyses, and (2) to allow for the exploration of sufficient spatial variation. We mapped the resulting odds ratios to visually explore spatial patterns.

Based on the observation that the spatial pattern of the OR values more of less coincides with sub-districts of Amsterdam, we decided to perform an additional stratified multilevel analysis by sub-district. This allows us to assess the associations more accurately than within GWR because of the limited number of observations in the local regressions. We restricted the stratified analysis to Nieuw-West where most participants reside.

## Results

Table 1 describes the characteristics of the study population in both ethnic groups. In general, no substantial differences in poor self-rated health, PCS and MCS were observed between Turkish and Moroccan participants. The two groups also had similar scores on most other characteristics although more Turkish participants were lower educated and had a little more difficulties in making ends meet.

Table 2 shows the average levels and standard deviations of own-group density, other-group density and ethnic heterogeneity by spatial scale for the two ethnic groups. Compared to Turkish participants, the residential environment of Moroccan participants was characterized by a higher share of co-ethnics. Levels and standard deviations of own-group density decreased with increasing buffer size, especially among Moroccans. Turkish participants had a higher percentage of Moroccans in their residential environment than vice versa. The difference between Turkish and Moroccan participants on the measures was approximately 10% points at all buffer distances. Levels and standard deviations of other-group density decrease with increasing buffer size, especially among Turkish participants. The level of ethnic heterogeneity of the residential environment of Moroccan and Turkish participants is comparable. Ethnic heterogeneity increases for buffers up to 500 m.

Table 3 shows the association of own-group density, other-group density and ethnic heterogeneity with self-rated health per ethnic group. Overall, own-group density and ethnic heterogeneity were not significantly related to self-rated health in both groups. For other-group density, a higher percentage of Turks in the neighbourhood was associated with higher odds of reporting better self-rated health among Moroccans. These results were consistent with more significant relations found for smaller buffers. Self-rated health of Turks was not significantly associated with higher density of Moroccans in the neighbourhood.

**Table 3:** Association of density of Moroccans, density of Turks and ethnic heterogeneity with better self-rated health, per ethnic group and spatial scale

Ethnic group	Moroccan	Turkish
	Standardised OR^a^ (CI^b^)	Standardised OR^a^ (CI^b^)
Density of Moroccans (%)		
Buffer50	1.01 (0.89; 1.14)	1.05 (0.94; 1.19)
100	1.08 (0.95; 1.23)	1.08 (0.95; 1.23)
150	1.10 (0.96; 1.26)	1.05 (0.92; 1.20)
300	1.09 (0.95; 1.25)	1.05 (0.92; 1.20)
500	1.09 (0.96; 1.24)	1.04 (0.92; 1.19)
750	1.11 (0.98; 1.26)	1.08 (0.96; 1.23)
1000	1.15 (1.02; 1.30)*	1.08 (0.96; 1.23)
Density of Turks (%)		
50	1.19 (1.07; 1.33)**	1.10 (1.00; 1.22)
100	1.16 (1.04; 1.30)**	1.06 (0.95; 1.18)
150	1.17 (1.04; 1.31)**	1.07 (0.96; 1.19)
300	1.15 (1.03; 1.30)*	1.05 (0.94; 1.17)
500	1.14 (1.01; 1.28)*	1.08 (0.97; 1.21)
750	1.14 (1.01; 1.28)*	1.09 (0.97; 1.22)
1000	1.16 (1.02; 1.31)*	1.07 (0.95; 1.21)
Ethnic heterogeneity		
50	0.98 (0.89; 1.09)	0.98 (0.89; 1.08)
100	1.03 (0.93; 1.15)	0.97 (0.88; 1.08)
150	1.03 (0.92; 1.15)	0.99 (0.89; 1.10)
300	1.10 (0.97; 1.24)	0.97 (0.86; 1.08)
500	1.15 (1.01; 1.31)*	1.03 (0.92; 1.16)
750	1.11 (0.97; 1.26)	1.06 (0.94; 1.20)
1000	1.14 (0.99; 1.31)	1.09 (0.96; 1.24)

* Significant at the 0.05 level

** Significant at the 0.01 level

^a^OR represents the standardised Odds Ratio (i.e. change in odds of having better self-rated health with one standard deviation increase in the predictor variable)

^b^CI represents 95% confidence interval

Table 4 shows the associations of own-group density, other-group density and ethnic heterogeneity with PCS and MCS per ethnic group. Among Moroccans a higher density of Turks within a 50 m buffer was significantly associated with a healthier PCS. Among Turks, higher ethnic heterogeneity was significantly associated with worse PCS at buffer sizes up to 300 m and with worse MCS from 150 to 500 m buffers.

**Table 4:** Association of density of Moroccans, density of Turks and ethnic heterogeneity with Physical and Mental Component Score per ethnic group and spatial scale

Ethnic group	Moroccan		Turkish	
	Standardised b^a^ (CI^b^)		Standardised b^a^ (CI^b^)	
	PCS	MCS	PCS	MCS
Density of Moroccans (%)				
Buffer50	0.01 (−0.04; 0.05)	0.03 (−0.02; 0.07)	0.01 (−0.04; 0.05)	−0.01 (−0.06; 0.04)
100	0.02 (−0.03; 0.07)	0.00 (−0.05; 0.06)	0.01 (−0.04; 0.06)	−0.02 (−0.07; 0.04)
150	0.01 (−0.04; 0.06)	0.00 (−0.05; 0.06)	0.01 (−0.04; 0.06)	−0.04 (−0.10; 0.01)
300	0.00 (−0.05; 0.05)	0.01 (−0.04; 0.07)	−0.01 (−0.06; 0.04)	−0.01 (−0.07; 0.04)
500	−0.00 (−0.05; 0.05)	0.01 (−0.04; 0.06)	−0.00 (−0.05; 0.05)	−0.03 (−0.08; 0.02)
750	0.01 (−0.04; 0.05)	0.00 (−0.05; 0.05)	0.01 (−0.03; 0.06)	−0.02 (−0.07; 0.03)
1000	0.01 (−0.04; 0.06)	0.00 (−0.05; 0.05)	0.02 (−0.02; 0.07)	−0.02 (−0.07; 0.04)
Density of Turks (%)				
50	0.04 (0.00; 0.08)*	0.01 (−0.03; 0.06)	0.01 (−0.03; 0.05)	−0.01 (−0.05; 0.03)
100	0.04 (−0.00; 0.08)	0.01 (−0.03; 0.06)	0.01 (−0.03; 0.05)	−0.01 (−0.06; 0.03)
150	0.04 (−0.00; 0.08)	0.01 (−0.04; 0.05)	0.01 (−0.03; 0.05)	−0.02 (−0.06; 0.03)
300	0.03 (−0.02; 0.07)	0.01 (−0.04; 0.05)	0.01 (−0.03; 0.05)	−0.02 (−0.07; 0.02)
500	0.01 (−0.03; 0.05)	0.01 (−0.04; 0.05)	0.02 (−0.02; 0.06)	−0.01 (−0.06; 0.03)
750	0.01 (−0.03; 0.05)	−0.01 (−0.05; 0.04)	0.02 (−0.02; 0.06)	−0.01 (−0.06; 0.04)
1000	0.01 (−0.04; 0.05)	−0.01 (−0.06; 0.04)	0.03 (−0.01; 0.08)	−0.02 (−0.07; 0.03)
Ethnic heterogeneity				
50	−0.02 (−0.05; 0.02)	−0.02 (−0.06; 0.02)	−0.06 (−0.09; −0.02)**	−0.01 (−0.05; 0.03)
100	0.00 (−0.04; 0.04)	−0.01 (−0.05; 0.03)	−0.05 (−0.09; −0.01)**	−0.02 (−0.06; 0.02)
150	0.00 (−0.04; 0.04)	−0.02 (−0.07; 0.02)	−0.04 (−0.08; −0.00)*	−0.05 (−0.09; −0.01)*
300	0.04 (−0.01; 0.09)	−0.01 (−0.06; 0.03)	−0.06 (−0.10; −0.02)**	−0.07 (−0.11; −0.02)**
500	0.05 (−0.00; 0.09)	0.00 (−0.05; 0.05)	−0.02 (−0.06; 0.03)	−0.06 (−0.10; −0.01)*
750	0.03 (−0.02; 0.08)	−0.02 (−0.07; 0.04)	−0.02 (−0.07; 0.02)	−0.05 (−0.10; 0.00)
1000	0.03 (−0.02; 0.08)	−0.01 (−0.06; 0.05)	−0.00 (−0.05; 0.04)	−0.03 (−0.08; 0.02)

* Significant at the 0.05 level

** Significant at the 0.01 level

^a^b represents the standardised regression coefficient (i.e. change in the dependent variable with one standard deviation increase in the predictor variable)

^b^CI represents 95% confidence interval

**Fig. 1:**
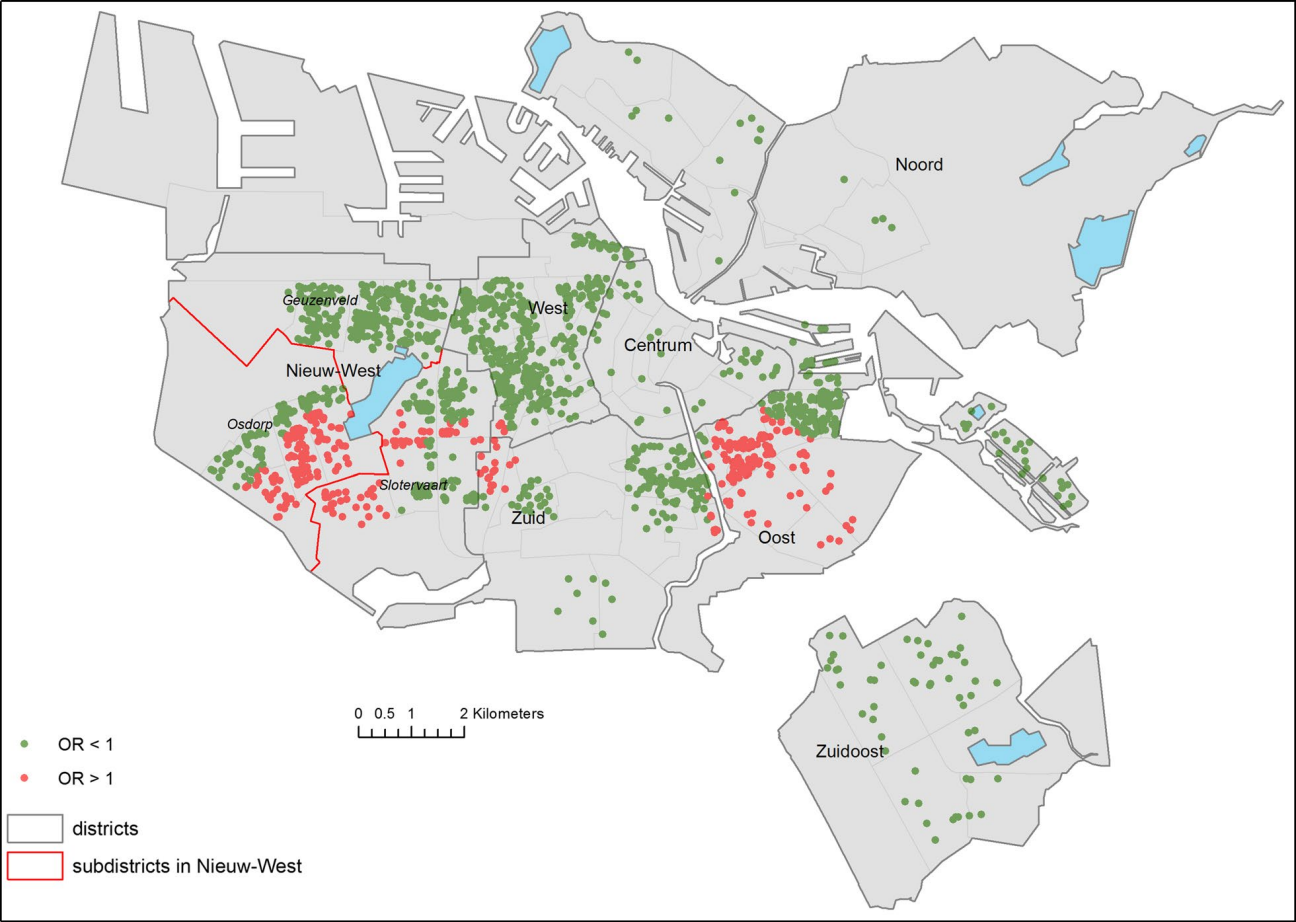
Association of percentage of Turks in buffers of 50 m with better self-rated health of Moroccans (odds ratios)

Based on the results of Table 3, we performed additional GWR-analyses to explore the spatial variation in the association of the density of Turks within 50 m buffers with self-rated health of Moroccans. The map in Fig. 1 shows some degree of spatial variation in this association, although most OR values were not significantly different from 1. In the district Nieuw-West, for example, the association of the density of Turks with self-rated health of Moroccans is more positive in the northern part of the district than in the southern part. In the district West mainly positive associations cluster and in East positive as well as negative associations were observed.

Table 5 assesses associations per sub-district in Nieuw-West. We stratified the additional MLR analyses by sub-district because the results of the GWR suggested variations at the level of sub-districts. We restricted the stratified analysis to Nieuw-West where most participants reside. Positive significant associations of density of Turks with self-rated health of Moroccans were found in the district Nieuw-West and mainly in the sub-district Geuzenveld. The density of Moroccans was significantly positively associated with self-rated health of Turks in Geuzenveld as well. For both groups, no significant association of own-group density with self-rated health was found in any district.

**Table 5:** Association of other- and own-group density within 50 m buffer with better self-rated health, per ethnic group and sub-district

Ethnic group			Moroccan		Turkish	
			Standardised OR^a^ (CI^b^)		Standardised OR^a^ (CI^b^)	
Density of			Turks (%)	Moroccans (%)	Moroccans (%)	Turks (%)
(Sub-)district	N Mor	N Tur				
Nieuw-West	1238	1365	1.16 (1.00;1.35)*	0.93 (0.76;1.12)	1.03 (0.86;1.23)	1.10 (0.96;1.26)
*Geuzenveld*	433	626	1.39 (1.04;1.86)*	0.86 (0.62;1.20)	1.32 (1.02;1.71)*	1.05 (0.86;1.29)
*Osdorp*	388	383	1.11 (0.85;1.45)	0.97 (0.67;1.40)	0.87 (0.59;1.28)	0.99 (0.74;1.32)
*Slotervaart*	416	356	1.02 (0.76;1.37)	1.01 (0.70;1.45)	0.81 (0.53;1.25)	0.98 (0.72;1.33)

* Significant at the 0.05 level

^a^OR represents the standardised odds ratio (i.e. change in odds with one standard deviation increase in the predictor variable)

^b^CI represents 95% confidence interval

## Discussion

In this study, we assessed associations between ethnic composition of the residential environment and self-reported health among people of Turkish and Moroccan origin living in Amsterdam. At the city-scale of Amsterdam, own-group density and ethnic heterogeneity were not associated with self-rated health for either Moroccan or Turkish participants. For Turks significant associations between ethnic heterogeneity and PCS and MCS were found, suggesting more negative health outcomes with increasing heterogeneity. With regard to other-group density, for Moroccans, greater density of Turks was significantly associated with higher odds of reporting better self-rated health and higher scores on PCS. Such associations were not found for Turks.

Additional geographical analyses suggest that the relationship between the density of the other group and self-rated health varies within Amsterdam. Associations were particularly observed in the sub-district Geuzenveld within the district Nieuw-West. In this specific area, other-group density is positively associated with self-rated health for both groups.

### Evaluation of data and methodology

A major strength of our study is that the HELIUS data provides a large number of participants from different ethnic groups and detailed health measurements and socio-demographic data. We further derived precise data about place of residence using the 6-digit postcode of the home addresses of the participants, and we accessed detailed socio-economic and demographic data from registries at the level of 6-digit postcodes. On average, 6-digit postcode areas in Amsterdam include no more than 10–20 households and are sized 50 by 50 m. The large number of participants and information on their precise place of residence enabled us to use advanced geographic techniques to explore varying associations within the city. The importance of using environmental variables at small spatial scales derives from the fact that most of the significant associations were found at small spatial scales. It suggests that no associations could have been demonstrated if the environmental characteristics of administrative areas were used because these areas may be too large to detect any health effects.

This study has some limitations as well. First, because buffers partly overlap, observations are not entirely independent. This results in a slight overestimation of significance levels. However, this problem of partial overlap applies particularly to larger buffers and less to smaller buffers, for which we found the most significant associations. Second, because our data are cross-sectional, our interpretations ought to refer to associations rather than to causal relationships. Nevertheless, we might interpret these associations as evidence for environmental influences on health. Reverse causality should refer to selective migration, which in our study would imply that healthy Moroccans would move to places with a lot of Turks or unhealthy Moroccans would leave such areas, which is not very plausible. Third, since we focused on two specific ethnic minority groups living in Amsterdam, our findings could possibly not be generalized to other populations or areas. Nonetheless, numerous large European cities have large migrant populations from Turkey and Morocco, so our findings might have relevance for these cities as well. Finally, PCS and MCS have not been validated among Turkish and Moroccan participants. However, these instruments have been positively validated across other cultures and countries [21, 22].

Our conceptualization of the residential environment, buffers, can be associated with two discussions in the research field, referred to as the ‘local trap’ [23] and the ‘residential trap’ [24]. The local trap refers to the question whether the local scale is the best scale for analysis and the residential trap refers to the neglect of other environmental context besides the residential context. Because we use different buffer sizes in our study, we could evaluate the local trap problem. In fact, the results imply that this problem is not so relevant on our cases, as the strongest associations were observed in the smaller buffers.

With regard to the residential trap, we admit that other environmental contexts are also important in determining people’s exposure to the own and other ethnic groups. To improve our understanding of the influence of other contexts, future research could try to combine different environmental contexts based on activity spaces. Activity spaces can be separated into domains such as a residential, transportation and work domain and for each domain the exposure to a certain environmental characteristic, for instance ethnic diversity, can be measured. Finally, the effects of the three exposure variables on a health outcome, such as mental health, can be assessed. This may yield new insights.

### Interpretation and comparison with previous studies

For Turks and Moroccans in Amsterdam we did not find associations of own-group density with self-rated health, PCS or MCS. These findings are not in line with ‘classic’ ethnic density theory which suggests better health if a high proportion of the own ethnic group lives in the neighbourhood. This positive influence on health is presumably due to increased social support and less discrimination if your own group lives around [25–28]. Several studies in the US and UK found effects of own-group density on health, sometimes positive [9, 29, 30], but sometimes negative [31–33]. However, similar to our results, Schrier et al. [12] found no association between own-group density and psychological distress for Surinamese, Turks, and Moroccans in the four largest Dutch cities (including Amsterdam).

The absence of an ethnic own-group density effect especially among Turks is surprising considering that the Turks are known as a group with a strong orientation towards their co-ethnics. It might be explained by segmentation within the Turkish community. Turks are a heterogeneous group, divided along often crosscutting lines associated with political, ethnic, religious and geographical differences [34]. Our measure for own-group density, which is based on the country of birth of the participants or their parents, may fail to comprehensively capture the own-group effects. If the subgroups would have lived entirely segregated, an own-group density effect for the Turkish participants might be expected. However, probably the subgroups live mixed because most of the Turks and Moroccans depend on social housing which means little room for own choice regarding place to live [35]. Unfortunately we miss the essential accurate information about the home location of subgroups for further examination.

The negative influence of ethnic heterogeneity on PCS and MCS among Turks accords with conclusions of Putnam’s study in the US [36] which suggested worse health conditions in heterogeneous neighbourhoods because of lower social capital in these neighbourhoods. For the Netherlands, Lancee and Dronkers [37] also found that more heterogeneous neighbourhoods are characterized by less social capital. However, our study did not find a negative effect of heterogeneity among Moroccans. Recently, it has been suggested that Putnam’s theory may not be generalizable to all ethnic groups [38], but depend on ethnic group identities and specific inter-group relations. In Amsterdam, for Turks a heterogeneous environment might be experienced as negative, because Turks are known as a group with a strong orientation towards (some of) their co-ethnics. Moroccans are known to have lower levels of co-ethnic cohesion [39]. Hence it could be suggested that Turks rely more on ‘bonding’ social capital (relations within the own group), while Moroccans may find it easier to link with other ethnic groups and thus rely on ‘bridging’ social capital (relations with other groups).

We found a positive influence of density of Turks on self-rated health of Moroccans. A previous study, based on a smaller survey among six ethnic groups in Amsterdam [13], found a negative influence of the density of Moroccans on self-rated health of Turks. Although the findings of the two studies are not identical, both imply that co-residence with Turks has no negative effect on self-rated health of Moroccans, and the Moroccans have no positive effect on Turks. This asymmetric relation might be explained by a lesser positive opinion of Turks towards Moroccans, partly because Moroccans are more stigmatized in Dutch politics and media than Turks [39, 40]. In such a context it is less favourable for Turks to be associated with Moroccans living in the same neighbourhood than vice versa. Another reason might be that Turks seems more oriented on the own group unlike Moroccans as already mentioned in the previous paragraph.

The positive influence of other-group density in the direct residential environment on self-rated health of both groups in Geuzenveld might be related to specific conditions in this area. Geuzenveld is an area with a relatively strong sense of community among Turkish and Moroccan inhabitants. Compared to other administratively defined areas in Amsterdam, Geuzenveld is smaller in size and the ethnic composition is dominated by only a few groups. Turks and Moroccans together comprise almost 50% of the population. This implies a relatively high degree of dependency and interaction between the two groups, with possibly stronger social support systems between these groups. This is reinforced by a low number of relocations and outmigration among ethnic groups in ethnic concentration areas such as Geuzenveld [41]. Moreover, the two groups may have forged stronger alliances with each other, given the context of strong tensions between ethnic minorities and those of Dutch origin in Geuzenveld [42], and relatively low socio economic position of Geuzenveld residents as compared to most other parts of Amsterdam [43].

Our findings may give some direction to policy aimed to improve urban health. The health effects of residential ethnic composition we found in this study reveal generally at small spatial scales and varied within the city. This suggests that to improve urban minority (self-rated) health, area-based local interventions are more appropriate than global city-wide interventions; health benefits will be larger if interventions are adjusted to specific problem locations. For instance, in areas with negative associations between other group density or heterogeneity and health, policy interventions could aim to increase interactions and social cohesion at the very local level.

## Conclusion

Our study suggests that in studies on the influence of neighbourhood ethnic composition on health three aspects are important. First, other-group density, the density of a specific ethnic group, deserves attention aside from common measures such as own-group density and ethnic heterogeneity. Additionally, it is important to use area measurements at small spatial scales. Finally, to improve our understanding of the underlying mechanisms, it might help to examine the spatial variation in the relationship within urban areas. The relationship between ethnic composition and health may depend on specific local factors influencing relations and ties between ethnic groups.

## Abbreviations

GIS: geographic information systems
MCS: Mental Component Score
PCS: Physical Component Score

## References

[1] Lee JJH, Guadagno L (2015). World Migration Report 2015. Migrants and cities: new partnerships to manage mobility.

[2] StatLine. Bevolking; generatie, geslacht, leeftijd en herkomstgroepering, 1 Januari 2015. http://statline.cbs.nl/. Accessed 20 Novembre 2015.

[3] Nielsen SS, Krasnik A (2010). Poorer self-perceived health among migrants and ethnic minorities versus the majority population in Europe: a systematic review. Int J Pub Health.

[4] Finch BK, Vega WA (2003). Acculturation stress, social support, and self-rated health among Latinos in California. J Immigr Health.

[5] Harris R, Tobias M, Jeffreys M, Waldegrave K, Karlsen S, Nazroo J (2006). Effects of self-reported racial discrimination and deprivation on Māori health and inequalities in New Zealand: cross-sectional study. Lancet.

[6] Lindström M, Sundquist J, Östergren PO (2001). Ethnic differences in self reported health in Malmö in southern Sweden. J Epidemiol Community Health.

[7] Reijneveld SA (1998). Reported health, lifestyles, and use of health care of first generation immigrants in The Netherlands: do socioeconomic factors explain their adverse position?. J Epidemiol Community Health.

[8] Mair C, Diez Roux AV, Galea S. Are neighbourhood characteristics associated with depressive symptoms? A review of evidence. J Epidemiol Community Health. 2008;62:940–6, 948 p following 946. doi:10.1136/jech.2007.066605. 10.1136/jech.2007.066605 18775943

[9] Pickett KE, Wilkinson RG (2008). People like us: ethnic group density effects on health. Ethn Health.

[10] Bécares L, Shaw R, Nazroo J, Stafford M, Albor C, Atkin K (2012). Ethnic density effects on physical morbidity, mortality, and health behaviors: a systematic review of the literature. Am J Public Health.

[11] Shaw RJ, Atkin K, Bécares L, Albor CB, Stafford M, Kiernan KE (2012). Impact of ethnic density on adult mental disorders: narrative review. Br J Psychiatry.

[12] Schrier AC, Peen J, de Wit MA, van Ameijden EJC, Erdem O, Verhoeff AP (2014). Ethnic density is not associated with psychological distress in Turkish-Dutch, Moroccan-Dutch and Surinamese-Dutch ethnic minorities in the Netherlands. Soc Psychiatry Psychiatr Epidemiol.

[13] Veldhuizen EM, Musterd S, Dijkshoorn H, Kunst AE (2015). Association between self-rated health and the ethnic composition of the residential environment of six ethnic groups in Amsterdam. Int J Environ Res Public Health.

[14] Damm AP (2009). Ethnic enclaves and immigrant labor market outcomes: quasi-experimental evidence. J Labor Econ.

[15] Damm AP (2014). Neighborhood quality and labor market outcomes: evidence from quasi-random neighborhood assignment of immigrants. J Urban Econ.

[16] Diez Roux AV, Mair C (2010). Neighborhoods and health. Ann N Y Acad Sci.

[17] Stronks K, Snijder MB, Peters RJG, Prins M, Schene AH, Zwinderman AH (2013). Unravelling the impact of ethnicity of health in Europe: the HELIUS Study. BMC Public Health.

[18] Stronks K, Kulu-Glasgow I, Agyemang C (2009). The utility of ‘country of birth’ for the classification of ethnic groups in health research: the Dutch experience. Ethn Health.

[19] Ware JE, Kosinski MMA, Keller SD (1996). A 12-Item Short-Form Health Survey: construction of scales and preliminary tests of reliability and validity. Med Care.

[20] Veldhuizen EM, Stronks K, Kunst AE (2013). Assessing associations between between socio-economic environment and self-reported health in Amsterdam using bespoke environments. PLoS ONE.

[21] Jenkinson C, Chandola T, Coulter A, Bruster S (2001). An assessment of the construct validity of the SF-12 summary scores across ethnic groups. J Public Health Med.

[22] Gandek B, Ware JE, Aaronson NK, Apolone G, Bjorner JB, Brazier JE, Bullinger M, Kaasa S, Leplege A, Prieto L, Sullivan M (1998). Cross-validation of item selection and scoring for the SF-12 Health Survey in nine countries: results from the IQOLA Project. J Clin Epidemiol.

[23] Cummins S (2007). Commentary: investigating neighbourhood effects on health-avoiding the ‘local trap’. Int J Epidemiol.

[24] Chaix B, Merlo J, Evans D, Leal C, Havard S (2009). Neighborhoods in eco-epidemiologic research: delimiting personal exposure areas. A response to Riva, Gauvin, Apparico, and Brodeur. Soc Sci Med.

[25] Smaje C (1995). Ethnic residential concentration and health: evidence for a positive effect?. Policy Polit.

[26] Halpern D (1993). Minorities and mental health. Soc Sci Med.

[27] Hunt MO, Wise LR, Jiguep MC, Cozier YC, Rosenberg L (2007). Neighbourhood racial composition and perceptions of racial discrimination: evidence from the black women’s health study. Soc Psychol Q.

[28] Bécares L. The ethnic density effect on the health of ethnic minority people in the United Kingdom: a study of hypothesised pathways. Doctoral dissertation. London: University College London; 2009.

[29] Veling MD, Susser E, van Os J, Mackenbach MD, Selten JP, Hoek HW (2007). Ethnic density of neighborhoods and incidence of psychotic disorders among immigrants. Am J Psychiatry.

[30] Das-Munshi J, Becares L, Dewey ME, Stansfeld SA, Prince MJ (2010). Understanding the effect of ethnic density on mental health: multi-level investigation of survey data from England. BMJ.

[31] LeClere F, Rogers R, Peters K (1997). Ethnicity and mortality in the United States: individual and community correlates. Soc Forces.

[32] White K, Borrell LN (2006). Racial/ethnic neighborhood concentration and self-reported health in New York City. Ethn Dis.

[33] Grady SC (2006). Racial disparities in low birthweight and the contribution of residential segregation: a multilevel analysis. Soc Sci Med.

[34] Inglis C, Akgonul S, De Tapia S (2009). Turks abroad: settlers, citizens, transnationals—introduction. IJMS.

[35] Van Praag C, Schoorl J, Crul M, Heering L (2008). Housing and Segregation. The position of the Turkish and Moroccan second-generation in Amsterdam and Rotterdam.

[36] Putnam R (2007). E pluribus unum: diversity and community in the twenty-first century the 2006 Johan Skytte Prize Lecture. Scan Polit Stud.

[37] Lancee B, Dronkers J, Hooghe M (2010). Ethnic diversity in neighborhoods and individual trust of immigrants and natives: A replication of Putnam (2007) in a West-European country. Social cohesion. Contemporary theoretical perspectives on the study of social cohesion and social capital.

[38] Abascal M, Baldassarri D (2015). Love thy neighbour? Ethnoracial diversity and trust re-examined. Am J Sociol.

[39] Slootman M, Duyvendak JW, Foner N, Simon P (2015). Feeling Dutch: the culturalization and emotionalization of citizenship and second-generation belonging in the Netherlands. Fear, anxiety, and national identity: immigration and belonging in North America and Western Europe.

[40] Identities Groenewold G, Relations Intercultural, Crul M, Heering L (2008). The position of the Turkish and Moroccan second-generation in Amsterdam and Rotterdam.

[41] Musterd S, de Vos S (2007). Residential dynamics in ethnic concentrations. Hous Stud.

[42] Boers J, van Marissing E, Slot J, Boutellier H (2012). Samenleven met verschillen: Signalering van spanningen en versterken van vertrouwen in Amsterdamse buurten.

[43] Municipality of Amsterdam. Gebiedsanalyse 2015. Retrieved from: http://www.ois.amsterdam.nl/pdf/2015_gebiedsanalyses_overkoepelende%20analyse.pdf.

